# COVID-19 Vaccines and Hyperglycemia—Is There a Need for Postvaccination Surveillance?

**DOI:** 10.3390/vaccines10030454

**Published:** 2022-03-16

**Authors:** Samson Mathews Samuel, Elizabeth Varghese, Chris R. Triggle, Dietrich Büsselberg

**Affiliations:** 1 Department of Physiology and Biophysics, Weill Cornell Medicine-Qatar, Education City, Qatar Foundation, Doha P.O. Box 24144, Qatar; elv2007@qatar-med.cornell.edu; 2 Department of Pharmacology, Weill Cornell Medicine-Qatar, Education City, Qatar Foundation, Doha P.O. Box 24144, Qatar; cht2011@qatar-med.cornell.edu

**Keywords:** COVID-19, diabetes, diabetic ketoacidosis, hyperglycemia, hyperosmolar hyperglycemic syndrome, SARS-CoV-2, COVID-19 vaccine

## Abstract

The COVID-19 vaccines currently in use have undoubtedly played the most significant role in combating the SARS-CoV-2 virus and reducing disease severity and the risk of death among those affected, especially among those with pre-existing conditions, such as diabetes. The management of blood glucose levels has become critical in the context of the COVID-19 pandemic, where data show two- to threefold higher intensive care hospital admissions and more than twice the mortality rate among diabetic COVID-19 patients when compared with their nondiabetic counterparts. Furthermore, new-onset diabetes and severe hyperglycemia-related complications, such as hyperosmolar hyperglycemic syndrome (HHS) and diabetic ketoacidosis (DKA), were reported in COVID-19 patients. However, irrespective of the kind of vaccine and dosage number, possible vaccination-induced hyperglycemia and associated complications were reported among vaccinated individuals. The current article summarizes the available case reports on COVID-19 vaccination-induced hyperglycemia, the possible molecular mechanism responsible for this phenomenon, and the outstanding questions that need to be addressed and discusses the need to identify at-risk individuals and promote postvaccination monitoring/surveillance among at-risk individuals.

## 1. Introduction

Coronavirus disease 2019 (COVID-19), caused by the SARS-CoV-2 virus, to date (6 March 2022), has affected over 445 million individuals and has claimed over 5.9 million lives worldwide [1]. Even after the identification of a new highly transmissible variant of concern, ‘B.1.1.529/Omicron’, on 26 November 2021 [2,3], due to currently available highly effective vaccines, Tozinameran/BNT162b2/Comirnaty (Pfizer/BioNTech), mRNA-1273/Spikevax (Moderna), AZD1222/ChAdOx1 nCoV-19/Covishield (University of Oxford/AstraZeneca), and Ad26.COV2.S/JNJ-78436735 (Janssen Vaccines and Prevention) [4], the world is in a better position to combat the reach and spread of the virus compared with the situation when the ‘Delta’ variant was rampantly spreading and increasing hospitalizations and deaths among affected individuals.

With over 10 billion doses of various COVID-19 vaccines (as of 6 March 2022; including booster doses) administered worldwide, the vaccines are deemed effective and safe with mild to moderate side effects (which include fever, fatigue, headache, muscle ache, chills, diarrhea and pain, redness, and swelling at the injection site) [5]. However, in rare cases, postvaccination anaphylactic reactions and other serious effects, such as vaccine-induced immune thrombotic thrombocytopenia (VITT) and related mortality, Guillain-Barré Syndrome (GBS), myocarditis, and pericarditis, were reported [5,6].

The increasing prevalence of diabetes, the occurrence of diabetes-associated complications, and hence the proper management of blood glucose levels have remained a persistent challenge for diabetic individuals and clinicians treating them. The management of blood glucose levels has become even more critical in the context of the COVID-19 pandemic. Several previously published data from diabetic COVID-19 patients showed increased COVID-19-related complications, disease severity, need for ICU admissions, and higher mortality rates compared with their nondiabetic counterparts [4,7]. Reports have also linked SARS-CoV-2/COVID-19 infection in diabetic and nondiabetic patients to acute pancreatitis and subsequent hyperglycemia or new onset of diabetes due to the direct or indirect cellular damage caused by the viral binding, accumulation, and replication in ACE2 receptor-expressing islets of the pancreas [4,7,8,9]. In this article, we intend to highlight and explore reports of possible COVID-19 “vaccine-induced” hyperglycemia (ViHG) and comment on the possible mechanism of ViHG and the need for postvaccination surveillance.

Data for this brief report were obtained by performing internet-based searches on ‘Google’ and ‘PubMed’ using a combination of keywords, such as “COVID-19 vaccine and hyperglycemia”, “COVID-19 vaccine and diabetes”, “COVID-19 vaccine and pancreatitis”, and “COVID-19 vaccine and VAERS”. The case studies mentioned in this report were published in 2021. Data regarding registered clinical trials related to COVID-19 vaccine and hyperglycemia/diabetes were obtained from ClincalTrials.gov (https://www.clinicaltrials.gov/) (search performed on 5 March 2022) using a primary search keyword (condition/disease): COVID-19 vaccine, and a secondary search keyword (other terms): hyperglycemia/diabetes. The search term “COVID-19 vaccine” was used in the VigiAccess website, the WHO Collaborating Centre for International Drug Monitoring (http://www.vigiaccess.org/) (accessed on 6 March 2022), to search for the number of reported COVID-19 vaccine-related “adverse drug reactions” pertaining to hyperglycemia/diabetes/pancreatitis.

## 2. Vaccine-Induced Hyperglycemia (ViHG)—Case Reports

Several instances of COVID-19 vaccination-induced hyperglycemia (ViHG) and related complications were reported [10,11,12,13]. Three diabetic individuals (one female and two males) presented with postvaccination hyperglycemia 1–6 days after receiving their first dose of the Covishield (AstraZeneca) vaccine [13]. The hyperglycemia persisted for about 30 days in the female and required a higher metformin dosage to achieve glycemic control [13]. The males achieved glycemic control in 3–15 days without additional antihyperglycemic intervention [13]. Another study reported acute hyperglycemia 20–36 days after administration of the first dose of the Covishield (AstraZeneca) vaccine in three middle-aged obese dyslipidemic male patients (two of whom were prediabetic) [11]. ViHG was reported after the administration of the mRNA vaccines, Tozinameran/BNT162b2/Comirnaty (Pfizer/BioNTech) and mRNA-1273/Spikevax (Moderna) [10,12]. A summary of their available pre- versus postvaccination metabolic profiles and key data is presented in Table 1. Interestingly, all the reported cases maintained a proper glycemic control before COVID-19 vaccination/the reported episode of hospitalization and/or ICU care (Table 1) [10,11,12]. In general, all patients presented with common osmotic symptoms of hyperglycemia (nocturia, polyuria, and polydipsia), and some reported significant weight loss, disorientation, and lightheadedness (Table 1) [10,11,12]. Irrespective of the diabetic status (nondiabetic/prediabetic/T2DM), previous history of COVID-19 infection, and type/dose of vaccine, all cases showed remarkably high blood glucose levels and HbA_1c_ levels at the time of hospitalization. The patients were diagnosed with hyperglycemic hyperosmolar syndrome (HHS) and/or diabetic ketoacidosis (DKA) and required saline and insulin infusion to manage the symptoms and condition (Table 1) [10,11,12]. Although HbA_1c_ levels measure the average blood glucose levels over 2–3 months, a sudden and significant increase in mean blood glucose levels can increase HbA_1c_ levels within 1–2 weeks and therefore recommend frequent testing in patients with uncontrolled diabetes [14,15]. Additionally, serum markers of inflammation (CRP, LDH, ferritin) were markedly higher in certain patients [12]. Data show that the treatment regimen was modified to an oral antidiabetic drug (metformin) postdischarge in most cases, and patients attained good glycemic control [10,12].

## 3. Possible Mechanism(s) of ViHG

While the reports discussed above point to a possible link between COVID-19 vaccine administration and resultant hyperglycemia and/or related complications, the rate of occurrence and the possible mechanism behind this phenomenon remain unclear. It is possible that the COVID-19 vaccine may have unmasked subclinical T2DM in predisposed individuals, such as in cases 1, 4, and 5 (Table 1), who were previously nondiabetic, and possibly in the prediabetic patients (cases 2 and 3; Table 1) [10,11,12]. However, it is also noteworthy that the blood glucose levels, postvaccination, were significantly elevated in the two patients (cases 6 and 7; Table 1) who were previously diagnosed with T2DM and in the three T2DM patients as reported by Mishra et al. [12,13]. However, while all these five T2DM patients were reportedly maintaining good blood glucose control through medication, diet, and exercise, as evident from their previous records, glycemic control was significantly offset following the COVID-19 vaccination [12,13].

As mentioned earlier, acute pancreatitis and subsequent hyperglycemia or new onset of diabetes, owing to the direct or indirect cellular damage of the ACE2 receptor-expressing pancreatic islets, was reported in SARS-CoV-2-infected patients [4,7,8,9]. Apart from acting as a binding site for the SARS-CoV-2 virus, the ACE2 receptor is also responsible for maintaining the critical balance required for the proper functioning of the renin–angiotensin–aldosterone system (RAAS) [4,7]. However, the functional downregulation of the ACE2 receptor (due to viral binding and the resulting hyperactivation of the AngII/AT1R axis) triggers proinflammatory signaling mechanisms and macrophage activation in target organs, such as the pancreas [7]. The hypercytokinemia (cytokine storm) that follows the exaggerated inflammatory and immune response (1) decreases pancreatic blood flow, (2) impairs β-cell function, and (3) increases cellular oxidative stress, damaging the pancreas (fibrosis), causing a reduction in synthesis and secretion of insulin and a decrease in insulin sensitivity in target tissues, leading to the increase in blood glucose levels [4,7,10].

**Figure 1:**
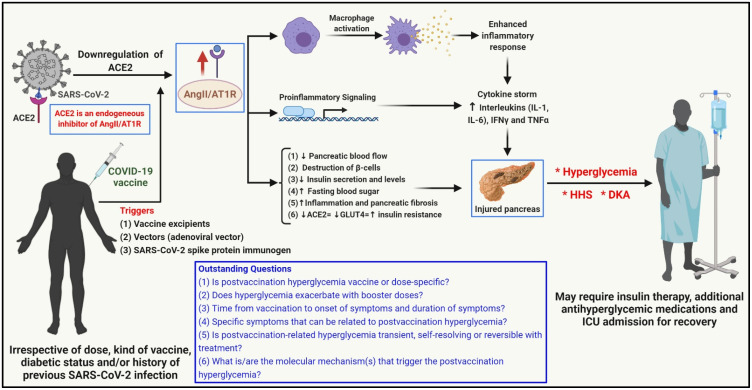
Possible mechanism of COVID-19 vaccination-associated pancreatic injury and hyperglycemia/hyperglycemic complications (figure adapted and modified from [7]). The angiotensin-converting enzyme 2 (ACE2) receptor, the key binding site for the SARS-CoV-2 virus, is a key regulator of the renin–angiotensin–aldosterone system (RAAS) and an endogenous inhibitor of the angiotensin II/angiotensin II type 1 receptor (AngII/AT1R) axis. SARS-CoV-2 binding to the pancreatic cells that express the ACE2 receptor and subsequent ACE2 downregulation diminish the inhibitory effect of ACE2 on the AngII/AT1R axis, which in turn leads to macrophage activation, upregulation of proinflammatory pathways, and hypercytokinemia (cytokine storm), culminating in pancreatic injury. It may be possible that vaccine excipients, vectors (adenoviral vectors), and the COVID-19 vaccine SARS-CoV-2 spike protein immunogen trigger similar mechanisms that cause pancreatic injury and subsequent hyperglycemia and hyperglycemic complications, such as hyperglycemic hyperosmolar syndrome (HHS) and diabetic ketoacidosis (DKA). Studies are warranted to address the outstanding questions in this area. Created with BioRender.com. * indicates potential postvaccination hyperglycemic scenarios.

Interestingly, pancreatic injury/acute pancreatitis (in some cases recurrent pancreatitis) were reported in individuals following COVID-19 vaccine administration and could be a possible cause for postvaccination hyperglycemia in the affected individuals [16,17,18,19]. There is a possibility that inflammatory mechanisms (elevation of proinflammatory cytokines, such as IL-1, IL-6, IFNγ, and TNFα) in response to a trigger (such as vaccine excipients), the adenoviral vector (as in the case of the Covishield vaccine), or the SARS-CoV-2 spike protein immunogen (derived from the vaccine) may culminate in pancreatic endocrine system damage and subsequently in an acute hyperglycemic milieu (Figure 1) [8,11,12]. It is also possible that some cases of ViHG may have been triggered by accidental IV injection with a resultant immune-mediated effect on the pancreas and subsequent pancreatitis. Myocarditis was reported in humans receiving vaccines for COVID-19 and, based on evidence from studies in rodents, potentially may have resulted following the inadvertent injection of the vaccine into a vein and a subsequent immune-mediated effect on cardiac tissue [20,21]. Although vaccine impurities (batch related) could be responsible for the vaccination-related inflammatory response, this is unlikely because the case reports presented patients who received different batches of the same vaccine or other kinds of COVID-19 vaccine [11].

## 4. Recommendations and Future Perspectives

Apart from the case reports of COVID-19 vaccine-related hyperglycemia and pancreatitis, as of 6 March 2022, VigiBase^TM^ (WHO Collaborating Centre for International Drug Monitoring) recorded 1464 cases of hyperglycemia, 1137 cases of diabetes mellitus, 25 cases of HHS, 398 cases of DKA, and 1610 cases of pancreatitis (including acute pancreatitis) that could be related to the COVID-19 vaccine administration (numbers are not specific for vaccine type or dose and do not represent a confirmed link) [22]. Among the billions of individuals who were vaccinated against COVID-19 and the ~3.3 million records on possible COVID-19 vaccine-related adverse drug reactions recorded on VigiBase^TM^, only 4634 records (~0.14%) were related to pancreatitis and/or hyperglycemia/diabetes/hyperglycemia-related emergencies [22].

Whether these vaccine-related adverse events are (1) rare occurrences, (2) missed due to lack of proper postvaccination surveillance, or (3) under-reported to avoid vaccine hesitancy remains unclear. Although Pfizer/BioNTech described a case of acute pancreatitis in their trial data, no other incidences of hyperglycemia or pancreatitis were reported in the trials conducted by any of the companies for their COVID-19 vaccines [17,18]. Interestingly, data from ClincalTrials.gov (https://www.clinicaltrials.gov/ (accessed on 6 March 2022)) (search performed on 6 March 2022; primary search keyword (condition/disease): COVID-19; secondary search keyword (other terms): vaccine, hyperglycemia) showed two clinical trials that are related to the effect of COVID-19 vaccines on blood glucose levels [23,24]. The clinical trial titled “mRNA based COVID-19 vaccine effects on blood glucose levels” (NCT04923386) intends to investigate the effect of mRNA-based COVID-19 vaccines on blood glucose levels (hyperglycemia) in patients with a history of both type 1 and type 2 diabetes by continuous glucose monitoring [23]. On the other hand, trial # NCT05233592 will investigate the “glycemic effects of COVID-19 booster vaccine in type 1 diabetes” by continuous glucose monitoring [24]. Although case series studies may not be appropriate to prove or disprove an association between COVID-19 vaccines and hyperglycemia/pancreatitis, the occurrence of COVID-19 vaccine-related hyperglycemia must not be overlooked, and continuous glucose monitoring is warranted in at-risk individuals (prediabetic, diabetic, previous history of COVID-19 disease) to further investigate this rare but potential vaccine-related adverse drug reaction to determine whether it can be avoided. Transparent communication remains the responsibility of scientists, clinicians, and public health organizations at the local, national, and international levels to create an overall public awareness regarding these rare but potential side effects of COVID-19 vaccines. At the same time, to avoid vaccine hesitancy, it is also essential that the overall benefits of vaccination versus very low risk of side effects are emphasized. Continuous postvaccination monitoring of blood glucose levels in every vaccinated individual will be time-consuming and needs significant effort and resources. However, the medical community and drug regulatory entities must be aware of pancreatitis and hyperglycemia as rare but possible COVID-19 vaccine-related severe adverse events (SAEs). Individuals presenting with postvaccination nocturia, polyuria, polydipsia, and abdominal pains/cramps should be examined accordingly, and the diagnosis and treatment regimens must be duly reported. At-risk individuals (prediabetic, diabetic, previous history of COVID-19 disease) must be identified and continuously monitored for the incidence of postvaccination hyperglycemia while encouraging postvaccination self-monitoring and reporting abnormal fasting and random blood glucose levels, among others. It could be helpful to extend the observation to larger segments of the population, particularly young people, to understand whether similar criticalities can occur among them.

Studies are warranted to address outstanding questions (Figure 1), such as (1) whether postvaccination hyperglycemia is vaccine or dose specific; (2) whether hyperglycemia exacerbates with booster doses; (3) time from vaccination to onset of symptoms and duration of symptoms; (4) specific symptoms that can be related to postvaccination hyperglycemia; (5) whether post-vaccination-related hyperglycemia is transient, self-resolving, or reversible with treatment; and (6) specific molecular mechanisms that trigger postvaccination hyperglycemia and whether this can be mechanistically related to SARS-CoV-2 infection-related onset of hyperglycemia.

## 5. Conclusions

The COVID-19 vaccines currently in use have undisputedly played the most significant role in combating the SARS-CoV-2 virus. This global initiative of promoting vaccinations must move forward to stop this pandemic, which has affected lives worldwide. Although developed, tested, and approved in record time, the COVID-19 vaccines are very effective and safe, and SAEs are extremely rare [10,11,12,16,18]. However, owing to the pressing need to control the spread of the virus, any long-term vaccination-associated SAEs that were initially missed (due to lack of long-term data) in the original vaccine trials will become evident in the long term from the emerging real-world data. Nonetheless, as the vaccination of the global population accelerates, there must be documentation and investigation of side effects, such as described for ViHG, so that, potentially, they can be predicted as regards the subjects most likely at risk and appropriate treatment can be initiated.

## Figures and Tables

**Table 1:** Published case reports of possible COVID-19 vaccination associated with hyperglycemia and hyperglycemic emergencies.

Gender/Age (Years)	Case 1 [11] Male/59	Case 2 [11] Male/68	Case 3 [11] Male/53	Case 4 [10] Male/58	Case 5 [12] Female/52	Case 6 [12] Male/59	Case 7 [12] Male/87
**BMI**	31.12	32.51	35.05	Data unavailable	32.5	27.7	26
**History of diabetes**	No	Prediabetic	Prediabetic	No	No	T2DM	T2DM
**Previous history of COVID-19 infection**	Data unavailable in case report	Data unavailable in case report	Data unavailable in case report	Data unavailable in case report	No	Yes	Yes
**Type of vaccine**	ChAdOx1 nCoV-19 vaccine (AstraZeneca)	ChAdOx1 nCoV-19 vaccine (AstraZeneca)	ChAdOx1 nCoV-19 vaccine (AstraZeneca)	Pfizer-BioNTech COVID-19 vaccine	Pfizer-BioNTech COVID-19 vaccine	mRNA-1273 Moderna vaccine	mRNA-1273 Moderna vaccine
**Symptoms**	Osmotic symptoms of hyperglycemia (polydipsia/polyuria)	Osmotic symptoms of hyperglycemia (polydipsia/polyuria)	Osmotic symptoms of hyperglycemia (polydipsia/polyuria)	Nocturia, polyuria, and polydipsia, severely dehydrated, disoriented, and significant weight loss	Polyuria, polydipsia, lightheadedness, and dysgeusia	Blurry vision, dry mouth, and polyuria	Fatigue, myalgia, weakness and altered mental status, polydipsia (despite increased fluid intake), confused, and poor appetite
**Dose of Vaccine—days to presentation/hospitalization**	First dose—21 days	First dose—36 days	First dose—20 days	Second dose—6 days	First dose—3 days	First dose—2 days	First dose—10 days
**Blood glucose levels (mg/dL) at time of hospitalization**	595	919	577	1253	1062	847	923
**C-peptide (normal range)**	235 pmol/L (370–1470 pmol/L)	561 pmol/L (370–1470 pmol/L)	377 pmol/L (370–1470 pmol/L)	1.1 ng/mL (1.10–4.40 ng/mL)	Data unavailable in case report	Data unavailable in case report	Data unavailable in case report
**HbA1c (%) (pre-episode)**	5.6 (~37 months prior)	6.5 (~24 months prior)	6.4 (~18 months prior)	Data unavailable (however, FBS ranged 74–120 mg/dL for past 3 years)	5.5–6.2 (~24 months prior)	7.5 (~8 weeks prior)	7.0 (~8 weeks prior)
* HbA1c (%) (during hospitalization)	14.1	14.7	17.1	13.0	12	13.2	Data unavailable in case report
**Diagnosis**	Hyperglycemic ketosis	Mixed HHS/DKA	DKA	HHS	T2DM and Nonketotic HHS	HHS	HHS and DKA
**Treatment/intervention**	Data unavailable in case report	Data unavailable in case report	Data unavailable in case report	In hospital: Hydration, insulin infusion, transitioning to subcutaneous multiple bolus of insulin. Further transitioning to glargine and insulin. Discharged: On insulin, which was tapered off, then discontinued and further simplified to only metformin.	In hospital: Hydration, insulin infusion, transitioning to subcutaneous basal bolus of insulin. Discharged: On glargine, lispro, and metformin, which was further simplified to only metformin and weekly dulaglutide.	In hospital: Hydration, insulin infusion, transitioning to subcutaneous basal bolus of glargine and lispro. Discharged: On glargine, metformin and sitagliptin, which was further simplified to only metformin.	In hospital: Hydration, insulin infusion, transitioning to subcutaneous basal bolus of insulin. Discharged: On glargine and metformin, which was further simplified to only metformin.
**HbA_1c_ (%) (postdischarge)**	Data unavailable in case report	Data unavailable in case report	Data unavailable in case report	Data unavailable in case report	6.7 (after ~7 weeks)	5.9 (after ~2 months)	Data unavailable in case report
**Blood glucose levels (mg/dL) (postdischarge)**	Data unavailable in case report	Data unavailable in case report	Data unavailable in case report	73 (FBS)	80–140	<140	Data unavailable in case report
**Postdischarge C-peptide (normal range)**	Data unavailable in case report	Data unavailable in case report	Data unavailable in case report	3.65 ng/mL (1.10–4.40 ng/mL)	1.45 ng/mL (1.10–4.40 ng/mL)	6.02 ng/mL (1.10–4.40 ng/mL)	Data unavailable in case report
**Study origin**	UK	UK	UK	USA	USA	USA	USA

Footnote: * HbA_1c_ levels measure the average blood glucose levels over 2–3 months; a sudden and significant increase in mean blood glucose levels can increase HbA_1c_ levels within 1–2 weeks and therefore recommend frequent testing in patients with uncontrolled diabetes [14,15]. DKA = diabetic ketoacidosis; HHS = hyperglycemic hyperosmolar syndrome; T2DM = type 2 diabetes mellitus.

## Data Availability

Not applicable.

## Abbreviations

ACE2	angiotensin-converting enzyme 2
AngII	angiotensin II
AT1R	angiotensin II type 1 receptor
COVID-19	coronavirus disease 2019
CRP	C-reactive protein
DKA	diabetic ketoacidosis
GBS	Guillain-Barré syndrome
GLUT4	glucose transporter 4
HHS	hyperglycemic hyperosmolar syndrome
ICU	intensive care unit
IFNγ	interferon gamma
IL-1/6	interleukin 1/6
LDH	lactate dehydrogenase
RAAS	renin–angiotensin–aldosterone system
SAE	severe adverse events
SARS-CoV-2	severe acute respiratory syndrome coronavirus 2
T2DM	type 2 diabetes mellitus
TNFα	tumor necrosis factor alpha
ViHG	vaccination-induced hyperglycemia
VITT	vaccine-induced immune thrombotic thrombocytopenia
WHO	World Health Organization
